# CAV-2 Vector Development and Gene Transfer in the Central and Peripheral Nervous Systems

**DOI:** 10.3389/fnmol.2019.00071

**Published:** 2019-03-29

**Authors:** Danila del Rio, Bertrand Beucher, Marina Lavigne, Amani Wehbi, Iria Gonzalez Dopeso-Reyes, Isabella Saggio, Eric J. Kremer

**Affiliations:** ^1^Institut de Génétique Moléculaire de Montpellier, University of Montpellier, CNRS, Montpellier, France; ^2^PVM, BioCampus, CNRS, INSERM, University of Montpellier, Montpellier, France; ^3^Department of Biology and Biotechnology “C. Darwin”, Sapienza University of Rome, Rome, Italy; ^4^Institute of Structural Biology, School of Biological Sciences, Nanyang Technological University, Singapore, Singapore

**Keywords:** adenovirus, CAV-2, coxsackievirus and adenovirus receptor, vectors, neurons, gene therapy, disease modeling, circuits

## Abstract

The options available for genetic modification of cells of the central nervous system (CNS) have greatly increased in the last decade. The current panoply of viral and nonviral vectors provides multifunctional platforms to deliver expression cassettes to many structures and nuclei. These cassettes can replace defective genes, modify a given pathway perturbed by diseases, or express proteins that can be selectively activated by drugs or light to extinguish or excite neurons. This review focuses on the use of canine adenovirus type 2 (CAV-2) vectors for gene transfer to neurons in the brain, spinal cord, and peripheral nervous system. We discuss (1) recent advances in vector production, (2) why CAV-2 vectors preferentially transduce neurons, (3) the mechanism underlying their widespread distribution via retrograde axonal transport, (4) how CAV-2 vectors have been used to address structure/function, and (5) their therapeutic applications.

## Understanding Structure and Function by Modification of Cells in the Central and Peripheral Nervous System

The human brain contains about 80 billion neurons and trillions of synapses. How a given subset of neurons influences behavior and cognition will never be completely understood. Nevertheless, many are trying to use less complex nervous systems to provide a rough blueprint of how these interactions could influence behavior. Chemical tracers have been used to map circuits and connections in the brain for decades (Vercelli et al., 2000; Carter and Shieh, 2015). Combining circuitry data with functional analyses based on the effect of ablation, diseases, infections, or injury of a given population of neurons also provides insight into the physiological role of some brain regions (Caeyenberghs et al., 2017). This foundation is now being built upon by the advent of gene transfer tools that modify cells at the injection site, modify neurons that synapse to those that are transduced at the injection site (via anterograde transsynaptic transport), or modify neurons that project into the injection site via retrograde transport of the vector.

Some of the current approaches to understand the function of neuronal subsets exploit chemo- and optogenetics (Adamantidis et al., 2015; Roth, 2016); they have further refined our understanding of the mammalian brain. Gene transfer tools that effectively target cells at the site of injection, as well as those in connected regions, are now available to deliver expression cassettes coding for chemo- and optogenetic proteins that extinguish or excite neurons. Moreover, the limitations in gene transfer efficacy are now frequently overcome by using novel and modified viral vectors.

## Clinical Gene Therapy

Gene therapy, i.e., using genetic material as a drug, can transiently or permanently endow target cells with novel or curative functions. Gene therapy approaches include, but are not limited to, gene replacement, gene correction, modifying mRNA stability, producing alternative gene products that reduce or increase the efficacy of cellular pathways, or endow cells with novel functions. The cornerstone in the optimization of *in vivo* gene transfer has been the steady improvement of vector design, delivery and expression kinetics. Viral vectors, which exploit natural uptake of viruses by cells, can be targeted to specific tissues by diverse means, including vector choice, vector tropism, the delivery mechanism, and/or modifying their expression parameters.

The *raison d’être* for clinical gene therapy targeting the central nervous system (CNS) is the unquantifiable impact on the patient and his/her entourage. Second comes the economic impact of neurodegenerative diseases, which often incurs costs for decades. Gene therapy to target neurodegeneration is particularly attractive given the insidious evolution of CNS diseases that deprive patients of their humanity. Here, neurodegeneration is defined by conditions that result in the loss of nerve structure/function that affects cognition, memory, or motor control. Among the hundreds of neurodegenerative disorders, considerable attention has been paid to the most common, in particular Parkinson’s and Alzheimer’s disease. Nonetheless, therapies for numerous brain diseases are in pre-clinical or Phase I/II/III stages. Examples include lysosomal storage disorders (e.g., Sly syndrome; Hunter’s, Batten’s disease), Huntington’s disease, childhood epilepsies (e.g., Dravet syndrome), leukodystrophies (e.g., Canavan disease), and motor control diseases (e.g., amyotrophic lateral sclerosis, spinocerebellar ataxia) (Piguet et al., 2017).

The major challenges for therapy of neurodegenerative disease are (1) targeting the correct population of cells (neurons, astrocytes, and/or microglia) in the desired structure(s), (2) repair of damaged or deteriorating neurons, (3) maintaining healthy and/or corrected cells alive in potentially toxic environments, (4) modifying enough target cells to make a clinical impact, (5) maintaining therapeutic levels of expression for decades, and/or (6) endowing the brain with self-repair capabilities. Self-repair will likely be the *Holy Grail* of brain gene therapy and may require the combination of vector-mediated and cell-based therapy. Indeed, because neurogenesis and astrogenesis continue throughout our lifetime, they could be combined with gene transfer for therapeutic approaches.

## *Panoply of Viral* Vectors

The number of viruses, or virus-like particles, that can be used to develop gene transfer tools is nearly limitless. Virologists divide vectors either by the type of genome they contain (single-stranded, double-stranded, segmented, linear or circular, RNA, or DNA) or by Families (which is used here). Some of the current vectors are made from the families *Adenoviridae* (adenovirus), *Retroviridae* (γ-retroviruses, HIV and other lentiviruses), *Poxviridae* (pox viruses, vaccinia virus), *Togaviridae* (α viruses/Semliki and SV-40), *Rhabdoviridae* (e.g., rabies virus), *Baculoviridae* (baculovirus), *Parvoviridae* (e.g., adeno-associated viruses), *Herpesviridae* (herpes simplex virus, cytomegalovirus, Epstein–Barr virus), and *Hepadnaviridae* (hepatitis B virus). The “virus-to-vector” transition includes using an unmodified capsid/envelope or altering it by borrowing pieces from another virus or a cell, or by adding moieties based on structure-based designs (Gao et al., 2005; Li et al., 2008; Kremer and Nemerow, 2015).

It is difficult to offer a balanced and critical analysis of the pros and cons of a viral vector without extensive experience in its production and *in vivo* use. While we have first-hand experience with adeno-associated viruses, γ-retroviruses, lentiviruses, baculoviruses, and picornaviruses, our expertise is with generating and using vectors derived from human and nonhuman adenoviruses. Therefore, here we provide an update of canine adenovirus type 2 (CAV-2) vectors for gene transfer to the central and peripheral nervous system (Junyent and Kremer, 2015).

### Adenoviridae

Adenovirus (Ad) infections occur in all human populations regardless of health standards (Lion, 2014). During repeated encounters, we generally develop multifaceted humoral and cellular immune responses (Perreau and Kremer, 2006; Kremer and Van De Perre, 2015; Mennechet et al., 2015; Eichholz et al., 2016; Tran et al., 2018). Nevertheless, many human Ad (HAd) types routinely establish persistent subclinical infection by mechanisms that are beginning to be identified (Zheng et al., 2016; Tran et al., 2018). As of 2018, approximately 90 types of Ad have been isolated from humans (Hage et al., 2017). They are broadly classed into 7 species (A–G), based on serology, agglutination characteristics, and genome sequences. Notably, species B and E arose via transmission from monkeys and great apes (Hoppe et al., 2015). There are also >300 nonhuman Ads that remain, for the most part, poorly characterized. The number of nonhuman Ads, isolated from mammals, reptiles, birds, and fish, will certainly continue to increase.

As a general rule, all Ads have an approximately 90 nm diameter, icosahedral, proteinaceous shell (i.e., they are nonenveloped) that encapsidates a linear, double-stranded DNA genome of 36 ± 8 kilobase pairs (Kremer and Nemerow, 2015). An increasing number of human and nonhuman Ads are being tested for their potential as a gene transfer tools (Duffy et al., 2018). The versatility of Ad genome and capsid parts allows one to create vectors for either short-term immunogenic responses (e.g., vaccines) or long-term stable transgene expression (e.g., for therapy for neurodegenerative diseases). It is worth noting that very few reports exist describing the efficacy of the majority of Ad vectors in the brain, spinal cord, or peripheral nervous system.

## Why a Vector From a Canine Adenovirus?

It is not surprising that vectors derived from viruses that generate a multi-faceted immune response in humans are not ideal candidates for clinical gene transfer. To reduce or circumvent immune-related drawbacks, numerous strategies have been used, including the induction of tolerance, immunosuppression, chemical and genetic modifications of the capsid (Nettelbeck et al., 2004; Lu et al., 2006; Kreppel and Kochanek, 2008; Toivonen et al., 2009; Prill et al., 2011). In the early 1990’s, Klonjkowski et al. (1997) initiated the creation of canine type 2 (CAdV-2 or more commonly referred to as CAV-2) vectors (Paillard, 1997). At that time, CAV-2 was the only nonhuman Ad that had been sequenced and produced as a vaccine for domestic dogs. An attenuated strain (Manhattan) of CAV-2 is still used as a vaccine against the more virulent CAV-1. Only in 1999 was a replication-defective CAV-2 vector isolated free of wild type CAV-2 (Kremer et al., 2000). Similar to most HAd vectors, CAV-2 was made replication-defective by deleting the early region 1 (E1). This codes for transactivating factors needed to upregulate viral gene expression and downregulate host cell genes (Horwitz, 1996; Berk, 2005). Thus, CAV-2 vectors must be propagated in CAV-2 E1-transcomplementing canine cells.

### Democratizing CAV-2 Vector Development – SLiCE and Dice With I-SceI

The development of replication-defective CAV-2 vectors was quite a challenge in the mid 1990’s. Similar to the human cell lines 293 and 911 (Fallaux et al., 1998; Shaw et al., 2002) that express the human Ad2 E1 region, we generated a canine cell line that expressed the CAV-2 E1 region, notably because CAV-2 does not propagate efficiently in human cells. While generating this canine cell line was relatively straightforward, selecting a clone for vector production was only possible when we created the vector.

**FIGURE 1:**
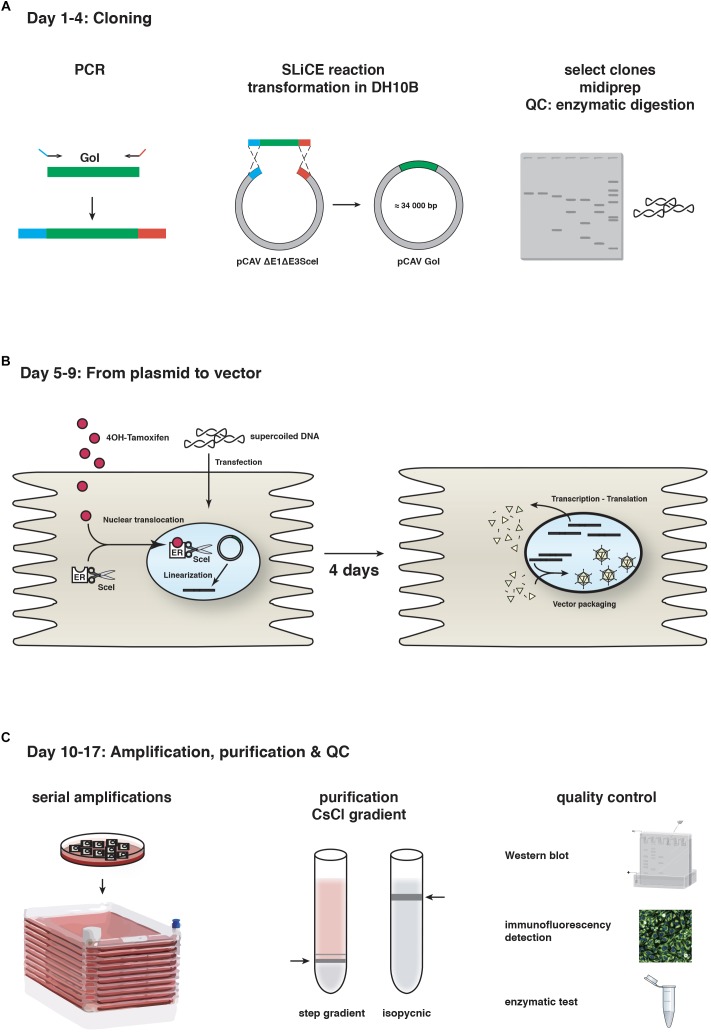
CAV-2 vector production. A schematic representation of the steps and timeline for **(A)** cloning using SLiCE, **(B)** vector generation from plasmid using SceI-expressing cells, and **(C)** vector amplification in cell factories, purification and quality control (QC). GoI, gene of interest.

At the time, recombinant vectors were created through homologous recombination between two DNA fragments transfected into transcomplementing cells (Kremer and Perricaudet, 1995). However, canine cells are notoriously difficult to transfect with linear, 32 kbp DNA fragments (efficiency typically < 3%). This precluded the use of homologous recombination in cells and in turn, vector isolation and production. The breakthrough in CAV-2 vector generation occurred when we adapted homologous recombination in bacteria, which is now routinely used to insert an expression cassette into a plasmid containing the HAd vectors. This strategy uses a shuttle plasmid together with a plasmid containing the Ad genome in *recAB*^+^-competent bacteria, typically BJ5183 cells. While homologous recombination in BJ5183 cells works for CAV-2 vector construction, it was ∼100-fold less efficient using the equivalent fragment from the human HAd type 5 (HAd5) genome. To circumvent this problem, we now use seamless ligation cloning extract (SLiCE) strategy (Zhang et al., 2012). Briefly, SLiCE was initially developed using an extract of the bacterial strain PPY, which contains λ prophage Red/ET recombination system, to mediate recombination between a DNA insert and a linearized vector. It is also possible to generate SLiCE extracts with the commonly-used bacterial strains DH5a, DH10b, XL10-gold or SURE2 (Motohashi, 2015). We find that SLiCE is an efficient, simple, inexpensive, and rapid method for cloning expression cassettes into a 32 kbp plasmid containing the CAV-2 genome. Expression cassettes are inserted directly into either the E1, the E3 (which codes for proteins involved in limiting the immune response to the infected cells and is dispensable for vector use), or the E1 and E3 regions in less than 1 week. Notably, SLiCE eliminates the need for the shuttle plasmid used in the commercially available “AdEasy system” (Figure 1).

The second step is generating viral vectors from the plasmid that contains the recombinant vector genome. As mentioned above, canine cells are poorly transfected with linear 32 kbp DNA fragments. To circumvent this, we adapted another technique based on the use of I-SceI activity in DKE1 cells (Ibanes and Kremer, 2013). We generated DKSce1 cells constitutively expressing I-SceI fused to the hormone binding domain of the oestrogen receptor (ER). I-SceI is a yeast endonuclease that recognizes an 18 bp sequence (Choulika et al., 1994). In the absence of the ER ligand 4-OH tamoxifen, the I-SceI-ER protein remains cytoplasmic, thereby avoiding potential damage to the cellular genome. The 18 bp I-SceI recognition site was inserted flanking the CAV-2 genome in the plasmid backbone, and freshly prepared supercoiled plasmid DNA increases transfection efficacy to > 50% in DKSce1 cells. 4-OH-tamoxifen-mediated nuclear translocation of the I-SceI-ER allows excision of the vector genome from the circular plasmid (Ibanes and Kremer, 2013). Restriction by I-SceI is a prerequisite because an Ad genome needs two free ends to initiate replication. This technical advance increased CAV-2 vector generation from plasmid DNA by <1,000-fold. The combination of SLiCE and I-SceI allows us to generate, under optimal conditions, a CAV-2 vector deleted in the E1 and/or E3 regions in 17 days (Figure 1).

### The Production and Advantages of Helper-Dependent CAV-2 Vectors

E1/E3-deleted CAV-2 vectors have numerous advantages: a cloning capacity of >7 kbp; can be purified in high titers (>10^13^ physical particles/ml) in a basic research laboratory; the highest ratio of infectious units/physical particles reported for any viral vector (>1:3) (Kremer et al., 2000); weak recognition by both the adaptive immune response in rats and pre-existing immunity in humans (Perreau and Kremer, 2005, Kremer, 2006; Perreau et al., 2007a,b). However, larger expression cassettes are needed in some cases. In these cases, helper-dependent (HD) CAV-2 vectors (Soudais et al., 2004) are useful. HD vectors lack all regions coding for viral proteins but retain the inverted terminal repeats (ITRs) and packaging signal (ψ) (Brunetti-Pierri and Ng, 2016). The 198 bp CAV-2 ITRs are needed for DNA polymerase to initiate genome replication. The 150 bp CAV-2 ψ (Soudais et al., 2001a) is bound by proteins that initiate the insertion of the linear genome into the capsid (Alba et al., 2011). HD vectors, also called high-capacity (HC), gutted, or gutless, can accommodate DNA inserts of up to 36 kbp. In most tissues, HD vectors increased the duration of transgene expression (Morral et al., 1998; Amalfitano and Parks, 2002; Kreppel and Kochanek, 2004; Brunetti-Pierri et al., 2007; Ariza et al., 2014; Cubizolle et al., 2014; Serratrice et al., 2014; Brunetti-Pierri and Ng, 2016). Given their characteristics, these vectors further improve *in vivo* safety and efficacy for long-term treatment of neurodegenerative diseases.

To generate HD vectors, cloning can be performed in *E. coli* via classic digestion/ligations, homologous recombination, or via SLiCE. Whether the 95–105% genome size limit for HAd5 vectors (i.e., minimum genome size of ∼34 kbp and a maximum of 38 kbp) applies to the 32 kbp CAV-2 genome and capsid is unknown. As in the case of E1-deleted CAV-2 vector generation, the HD genome is transfected into DKE1 cells that are co-infected with an E1-deleted “helper” vector, which provides the viral proteins in *trans* during the 36 h propagation cycle. While both HD and helper genomes replicate, helper genome packaging is prevented by flanking its ψ with the 34 bp *lox* sequences together with expression of Cre recombinase by the cells or the helper vector (Kochanek et al., 1996; Morsy et al., 1998). *Ipso facto*, Cre excises the helper’s ψ, leading to preferential packaging of the HD vector genome.

Unfortunately, constitutive expression of Cre recombinase in DKE1 cells (DKCre cells) decreases cell viability, protein expression by the E1 region, and production of HD CAV-2 vectors (Fernandes et al., 2015a; Simão et al., 2016). Multiple amplification steps are needed to produce HD CAV-2 vectors, which also hampers robust production and in turn the availability of high quality preps. This led us to analyze the progression of the HD vector propagation cycle (Fernandes et al., 2015b). Paulo Fernandes found that the helper genome replicates faster during HD vector production compared to E1-deleted vectors alone. This is mirrored by increased expression of the CAV-2 polymerase, pre-terminal protein, and structural proteins. While genome packaging resembles that of E1-deleted vectors, more immature capsids are generated during HD production. This leads to a fourfold increase in the physical-to-infectious particles ratio, as well as augmented autophagy and cell death, which further compromises productivity. One potential approach to improve HD CAV-2 production is a helper vector with a floxed ψ, which also expresses a transcriptionally-regulated CreERT2 cassette (Gonzalez-Aparicio et al., 2011). This vector would allow the use of DKSceI cells for HD CAV-2 vector production and potentially increase both production and the infectious units/physical particle ratio.

## Cav-2 Preferentially Transduces Neurons Due to Car Expression

The tropism of a virus is usually associated with the clinical symptoms. However, the tropism of viral vectors does not *a priori* mimic that of their virus of origin. Similar to the tropism of HAd5, CAV-2 is thought to preferentially infect the upper respiratory track in Canidae and Ursidae. In dogs, CAV-2 causes a mild disease called “kennel cough” (Wright et al., 1972). In the case of a vector, cellular and tissue targets are a function of multiple factors: (1) the mode of injection (e.g., intravenous, subcutaneous, intradermal, intramuscular, intranasal, intracerebral, intrathecal…), (2) host physiology (e.g., when the blood brain barrier closes), (3) capsid modifications, (4) interaction with extracellular components (5) fluid flow dynamics, and other factors. Upon intracerebral and intramuscular injection, CAV-2 vectors preferentially transduce neurons (Soudais et al., 2001b). When a CAV-2 vector expressing GFP (CAVGFP) was placed in the olfactory cavity, which is predominantly lined with columnar epithelial cells, sensory olfactory neurons were preferentially transduced. Injection in the hindleg muscle in newborn mice led to poor transduction of myofibers but a surprising specificity for the innervating motoneurons via retrograde axonal transport (Soudais et al., 2001b, 2004; Salinas et al., 2017). Similarly, following injection into the highly innervated diaphragm, few muscle cells were transduced but a significant number of neuromuscular junctions were GFP^+^, again demonstrating a preference for motoneurons and axonal retrograde transport of CAV-2 (Soudais et al., 2001b). When injected into the rodent brain parenchyma, CAV-2 vectors preferentially transduced neurons at the site of injection, as well as the neurons that project into this area (see Box 1). Following injection in the striatum, dopaminergic neurons of the *substantia nigra pars compacta* (SNpc), thalamic neurons, and cortical neurons (layer IV) of the ipsilateral and contralateral neocortex are transduced (Soudais et al., 2001b; Hnasko et al., 2005; Kremer, 2005; Bru et al., 2010).

We also injected a HD CAV-2 vector expressing GFP (HD-GFP) in the *Microcebus murinus* caudate nucleus (Mestre-Francés et al., 2018). *M. murinus*, commonly called the gray mouse lemur, is small nocturnal primate from Madagascar, whose brain structure and organization are comparable to that of the human brain. *M. murinus* have been bred in captivity since the 1960s, with lifespan of ≥ 10 years. This primate is increasingly used to study aging, Alzheimer’s disease (including amyloid-β vaccination) (Trouche et al., 2009), and identification of cognitive deficits (Kraska et al., 2009; Trouche et al., 2010). Due to captive breeding programs, *M. murinus* is one of the few primates allowed by current European regulations for research. After HD-GFP injection in the striatum, GFP^+^ somata and processes were found at the injection site, throughout the frontal and occipital cortex in both hemispheres, in the *SNpc* of both hemispheres, and in the ipsilateral basal nuclei of Meynert. The dense GFP signal surrounding the injection site was consistent with transduction of striatal neurons and afferent axons from other brain areas. In some animals, the vector leaked into the ventricles/cerebral spinal fluid and transduced SOX2^+^ cells lining the lateral ventricles. It is unknown if these SOX2^+^ cells were equivalent to the neural precursor cells infected by CAV-2 in the mouse brain (Salinas et al., 2017).

**BOX 1 |** Recent examples of CAV-2 vector use.

• The Adan lab used the same tools to identify the ventral tegmental area (VTA) to nucleus accumbens pathway. In some cases, this approach can circumvent the need to implement glass fibers for optogenetic stimulation and complements the use of transgenic Cre mice (Boender et al., 2014).
• Using a similar approach, Carter et al. (2013) demonstrated that the neural circuit from the parabrachial nucleus to the central nucleus of the amygdala is involved in the suppression of appetite.
• Lerner et al. (2015) used CAV-2 vectors to identify two distinct nigrostriatal DA circuits with differing in inputs, outputs, biophysical properties, and environmental information representations. Both circuits independently control information representations streaming through *SNpc* and each provides a generalizable framework for brain-wide mapping of diverse populations of neurons defined by multiple independent types of features (Lerner et al., 2015).
• The Maren lab showed that medial prefrontal cortex-thalamic nucleus reuniens circuits inhibit the expression of Pavlovian fear memories in rats, a function that influences adaptive emotional regulation (Ramanathan et al., 2018).
• By using CAV-2 vectors and DREADD-mediated inhibition of laterodorsal tegmentum excitatory cholinergic inputs to the VTA, the Barik lab in Marseilles characterized a neuro-circuitry implicated in depressive-like disorders (Fernandez et al., 2018).
• The Huberman lab explored how our internal state is merged with our visual perception of an impending threat to drive an adaptive behavioral response. They showed that the nucleus reuniens, nuclei of the ventral midline thalamus, and the xiphoid nucleus (Xi) are implicated in controlling behavioral responses to visual threats (Salay et al., 2018).
• In Bordeaux, Senn et al. (2014) showed using CAV-2 vectors and optogenetics that dorsal medial prefrontal cortex that projects to the lateral and ventrolateral periaqueductal gray circuits are necessary for discriminating a previously threatening context from a neutral context.
• Using CAV-2 vectors and chemogenetic silencing of the lateral hypothalamus-lateral habenula pathway, the Mameli lab showed that aversive stimuli such as foot-shocks drive hypothalamus-to-habenula excitation to promote escape behavior in mice (Lecca et al., 2017).
• The Stuber lab showed that in addition to intra-cortical connectivity, prefrontal cortical projection neurons innervate subcortical structures that contribute to reward-seeking behaviors. Using CAV-2 retrograde transport for bidirectional optogenetic manipulation of these neurons allowed them to demonstrate that stimulation of corticostriatal neurons promotes conditioned reward-seeking behavior after learning. By contrast, activity in corticothalamic neurons suppresses both the acquisition and expression of conditioned reward seeking (Otis et al., 2017).
• The locus coeruleus (LC) projects to almost the entire neuro-axis and plays a role in learning and memory, pain, motivation, strategic behavior, and arousal. The LC is the principal noradrenergic nucleus in the CNS and is the main source of noradrenergic innervation to the spinal dorsal horn, forming part of an analgesic circuit (Li et al., 2016; Hirschberg et al., 2017). It was unclear whether the LC acts functionally as a single global effector or as discrete modules. Specifically, while spinal-projections from LC neurons can exert analgesic actions, it was unknown whether they can act independently of ascending LC projections. The Pickering lab unraveled this dichotomy using CAV-2 uptake at axon terminals and a pharmaco-selective actuator module (PSAM) to selectively target LC neurons with spinal (LC→SC) or prefrontal cortex (LC→PFC) projections (Li et al., 2016; Hirschberg et al., 2017). Activation of the LC→SC module produced robust, lateralized anti-nociception while activation of LC→PFC produced aversion. In a neuropathic pain model, LC→SC activation reduced hind-limb sensitization and induced conditioned place preference. By contrast, activation of LC→PFC exacerbated spontaneous pain, produced aversion and increased anxiety-like behavior.
• Still in the LC, the Johansen lab examined how the circuit and neural-coding features of this neuromodulatory system regulates aversive emotional learning and behavioral flexibility in rats. They described a modular organization containing distinct neural projection patterns and coding properties for flexible specification of opposing behavioral learning states. An amygdala-projecting group promoted aversive learning, while a medial prefrontal cortex-projecting group extinguished aversive responses (Uematsu et al., 2017).
• The Nir lab examined how LC activity modulates sensory-evoked awakenings, testing whether reduced LC activity mediates sensory disconnection occurring in sleep. Optogenetic LC excitation using CAV-2-mediated ChR2 modulated arousal as shown by sleep-wake transitions, EEG desynchronization, and pupil dilation. Sounds presented on a background of weaker LC excitation (not awakening by itself) led animals to wake up frequently. Next, Hayat et al. (2019) silenced LC activity using a CAV-2 vector harboring a soma-targeted anion-conducting channelrhodopsin (stGtACR2) under the control of the PRS promoter and showed that it effectively silences LC activity and constricts pupils. Brief LC silencing around auditory stimulation reduced sound-evoked awakenings, showing that LC activity is both necessary and sufficient for modulating sensory-evoked arousal threshold (Hayat et al., 2019).
• Using CAV-2 vectors, targeted lesion, optogenetic, and chemogenetic stimulation of central amygdala of mice, the de Araujo lab identified coordinated circuits emanating from the central amygdala that control the efficiency of prey capture and the ability to deliver fatal bites to prey. Coordinated control of cervical and mandibular musculatures was mediated by a central amygdala projection to the reticular formation in the brainstem. By contrast, prey pursuit was mediated by projections to the midbrain periaqueductal gray matter (Han et al., 2017). The de Araujo lab also showed how the gut-brain neuronal circuitry regulates of motivational and emotional states. Using CAV-2 vector to infect gut-innervating vagal sensory neurons and optogenetics they found that right, but not left, vagal sensory ganglion activation sustained self-stimulation behavior, conditioned flavor and place preferences, and induced dopamine release from *SNpc* cells (Han et al., 2018).
• Asokan et al. (2018) examined how the layer 5 cortical neurons coordinate integrative auditory processing and adaptive behaviors. Using CAV-2 vectors they showed that auditory corticofugal neurons that innervate the inferior colliculus have widespread targets throughout the forebrain.
• The Tye lab has used CAV-2 vectors to identify and characterize several pathways (Allsop et al., 2014; Tye, 2014; Namburi et al., 2015; Beyeler et al., 2016; Vander Weele et al., 2018). These pathways include the medial prefrontal cortex projections to the dorsal periaqueductal gray (Vander Weele et al., 2018); the basolateral amygdala neurons during the retrieval of associative memories via synapse in the nucleus accumbens, the central amygdala, or ventral hippocampus (Namburi et al., 2015; Beyeler et al., 2016).
• At the Friedrich Miescher Institute for Biomedical Research in Basel, the Arber lab characterized motor collateral organization between the spinal cord and neurons in the brainstem. Pivetta et al. revealed a widespread and diverse network of spinal dual-axon neurons, with coincident input to forelimb motor neurons and the lateral reticular nucleus in the brainstem (Pivetta et al., 2014; Ruder et al., 2016). The Lüthi lab functionally characterized the connections of basal nucleus of the amygdala – medial prefrontal cortex (Vogel et al., 2016) and in particular the prelimbic and infralimbic subdivisions in fear generation and extinction.
• The Grinevich lab used CAV-2 vectors to separate magno- versus parvocellular oxytocin neurons and hypothalamic paraventricular neurons projecting to the supraoptic nuclei, and spinal cord deep laminae of L5 projections to cell bodies of oxytocin neurons in the paraventricular nuclei and their axonal projections in close proximity to somas and dendrites of magnocellular oxytocin neurons of the supraoptic nuclei (Eliava et al., 2016).
• The Arenkiel lab showed that the arcuate nucleus, receives cholinergic, and noncholinergic diagonal band of Broca projections to regulate appetite (Herman et al., 2016).
• The Chester lab used the retrograde transport of CAV-2 from the central nucleus of the amygdala and the dorsal reticular formation in the medulla to the parabrachial nucleus to help characterize a brainstem circuit that controls escape responses to select noxious stimuli (Barik et al., 2018).

In the *M. murinus* brain, the total number of GFP^+^ neurons at 6 months was equivalent to that at 2 weeks, demonstrating that the vectors led to long-term expression of a potentially immunogenic protein (GFP) (Mestre-Francés et al., 2018). These authors also quantified TH^+^/GFP^+^ neurons in the *SNpc* following vector deposit at a single coordinate in the caudate nucleus, and found a transduction efficacy of approximately 70% in the 3,000 TH^+^ neurons/hemisphere. Thus, these data demonstrate that in the primate brain CAV-2 vectors preferentially transduce neurons, are transported to afferent structures, and allow stable expression of a foreign protein.

## Cav-2 Is Retrogradely Transport (From the Axon Tip to the Soma)

As in the rodent brain (Zussy et al., 2016), expression of the coxsackievirus and adenovirus receptor (CAR) is restricted to neurons in the *M. murinus* brain parenchyma (Salinas et al., 2013; Mestre-Francés et al., 2018). While the transduction profile in the *M. murinus* brain is similar to that of the rodent brain, efficacy is globally better. CAV-2 infects neurons by binding to CAR at axon terminals (Salinas et al., 2009, 2010), however, the density and distribution of CAR along the surface of axons has not been extensively characterized. In most regions of the mouse brain, anti-CAR staining appears as small puncta along axons and dendrites (Zussy et al., 2016). Moreover, CAR is found in the presynapse fraction of synaptosome preparations from adult mouse, *M. murinus* and human brains (Zussy et al., 2016). While CAV-2 can efficiently enter a neuron via presynaptic termini, other entry sites are possible, but the efficacy is unknown (Schwarz and Luo, 2015).

**FIGURE 2:**
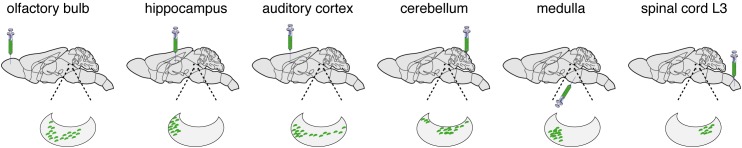
LC transduction. While working with the laboratories of Liqun Luo [100], Tony Pickering (Li et al., 2016; Hirschberg et al., 2017), and Johan Johansen (Uematsu et al., 2017), we have used the retrograde transport of CAV-2 vectors to characterize the connections and functions of the LC. Here we present some of the results showing LC connectivity as determined by CAV-2 retrograde transport. The green syringe shows where CAVGFP was injected. The kidney shaped blow up represents the LC and green dots are GFP positive soma.

Another aspect about CAV-2 vectors is that the preference for neuronal subtypes is not fully characterized. While CAV-2 infects many classes of neurons, such as motor, sensory, parasympathetic, GABAergic, cholinergic, norepinephrine (NE), and dopamine (DA) neurons (Figure 2 and Box 1; Hnasko et al., 2006; Salinas et al., 2009; Beier et al., 2015; Schwarz et al., 2015; Li et al., 2016; Uematsu et al., 2017), some neurons may not express CAR and are therefore transduced less efficiently if at all. A potential example of this is the comparison of CAV-2 vectors to an engineered AAV vector (AAV-retro) selected for its capacity for *in vivo* retrograde transport from the mouse basal pontine nuclei (Tervo et al., 2016). While the study provided no information with respect to dose, volume, number of animals, or controls, they concluded that CAV-2 vectors poorly infected these neurons and therefore did not induce significant cortical expression of GFP in Rosa26-LSL-H2B-eGFP mice after injection in the basal pontine nuclei.

Axonal transport is essential for neuronal homeostasis, as its impairment can be linked with neurodegenerative disorders (Hinckelmann et al., 2013; Millecamps and Julien, 2013; Gibbs et al., 2015; Grosch et al., 2016). Some viruses, including rabies, herpes simplex type I, and poliovirus, as well as tetanus toxin, use axonal transport to access the soma of neurons in the CNS (Salinas et al., 2010). Live-cell imaging and cell biology approaches allowed us to characterize the mechanisms regulating CAV-2 entry and transport in primary rodent motor neurons (Salinas et al., 2009; Henaff et al., 2011; Simão et al., 2015). CAV-2 trafficking occurs in pH neutral endosomes, which allows long-range transport in an environment that precludes pH-induced conformational changes of the capsid and endosomal escape. Interestingly, tetanus toxin and other viruses, along with neurotropic factors and their receptors, are transported in these endosomal structures (Salinas et al., 2010; Schmieg et al., 2014).

## How Cav-2 Has Been Used for Mps Therapy and to Model Neurodegenerative Disorders

### Treating Mucopolysaccharidoses Type IIIA and VII

Mucopolysaccharidoses (MPSs) are a group of rare, autosomal recessive disorders caused by deficiencies in the catabolism of glycosaminoglycans (GAGs) (Mehta and Winchester, 2012). Several MPSs are associated with neuropathologies presenting variable clinical symptoms (Eto and Ohashi, 2002). For MPS brain therapy, the majority of cells do not need to be transduced by a therapeutic vector due to the phenomenon of cross-correction (Neufeld, 2011). Nevertheless, transduced cells must be dispersed throughout the brain to generate local factories that secrete enzymes. Thus, multiple injections throughout the brain must be combined with a vector that is capable of widespread brain distribution. For these reasons, CAV-2 vectors are ideal candidates for gene transfer. Indeed, CAV-2 vectors have been tested for their ability to improve neuropathological changes associated with MPS IIIA and MPS VII (Lau et al., 2010; Ariza et al., 2014; Cubizolle et al., 2014; Serratrice et al., 2014).

Neonatal administration of CAV-2 vectors harboring a N-sulfoglucosamine sulfohydrolase expression cassette produced both dose-dependent and widespread transgene expression that persisted for at least 20 weeks and prevented memory and learning deficits in mice (Lau et al., 2010). By contrast, introduction of the same vectors in the thalamus and ventricles of adult MPS IIIA mice resulted in limited duration of N-sulfoglucosamine sulfohydrolase expression (Lau et al., 2012). There are several potential reasons for this: the adult MPS IIIA mouse brain is primed by damage-associated molecular pattern molecules (Yang et al., 2017) and GAGs that induce chronic inflammation and likely “trained immunity” (Mehta and Winchester, 2012; Saeed et al., 2014; Netea et al., 2016; Netea and van der Meer, 2017). This inflammatory-primed environment would render vector injection less benign and the detection of a pathogen-associated molecule, like the double-stranded DNA genome of a vector in the cytoplasm, would likely amplify an immune response.

This hypothesis is supported by the work from Ariza et al. (2014), who also showed that transient immune suppression dramatically improves the duration of transgene expression in the MPS VII mouse brain. MPS VII is caused by deficient ß-glucuronidase activity, which results in the partial degradation of chondroitin sulfate, dermatan sulfate, heparan sulfate, and gangliosides (Ray et al., 1999; Heuer et al., 2001). We demonstrated that a CAV-2 vector containing a ß-glucuronidase expression cassette restores global ß-glucuronidase activity, reduces GAG accumulation, and corrects both the enlarged storage vesicle and irregular lysosome morphology in the brains of MPS VII mice and the ∼200-fold larger MPS VII dog (beagle) brain (Ariza et al., 2014; Cubizolle et al., 2014). Equally relevant, this approach improved cognitive functions of MPS VII mice (Ariza et al., 2014). In contrast to a study using AAV vectors for MPS I/III therapy (Ellinwood et al., 2011), transient immunosuppression was sufficient when using HD CAV-2.

### Using CAV-2 Vectors to Model Parkinson’s Disease in Nonhuman Primates

Vector-mediated gene transfer can also be used to better understand neurodegenerative diseases. Due to the efficient infection of DA neurons in the *SNpc* following injection in the striatum (Soudais et al., 2001b, 2004; Hnasko et al., 2005, 2006; Mestre-Francés et al., 2018), Parkinson’s disease modeling is an attractive target for CAV-2 vectors. Like the NE neurons in the *locus coeruleus*, CAV-2 vectors can transduced > 90% of the rat or 70% of *M. murinus* DA neurons in the *SNpc* following injection into striatum (Soudais et al., 2001b, 2004; Mestre-Francés et al., 2018). Vector-mediated gene transfer can complement transgenic rodents and/or drug-induced disease models that have been invaluable, yet imperfect, for unraveling the mechanism of numerous brain and systemic disorders. The general consensus is that the lack of robust Parkinson’s disease animals reproducing its complex characteristics hampers progress in both the understanding of pathogenic mechanisms and identification of therapies. MPTP (1-methyl-4-phenyl-1,2,3,6-tetrahydropyridine)-induced Parkinson’s disease in primates is often used to test the efficacy of therapeutic approaches. However, MPTP induces acute and toxic injury to DA cells and poorly mimics the progressive course of Parkinson’s disease. In addition, MPTP injections do not lead to the definitive Parkinson’s disease pathological hallmarks of α-synuclein (α-syn) aggregates and Lewy body formation (Beal, 2010). One approach is to express LRRK2^G2019S^, the most common dominant-negative mutation in patients with familiar and sporadic Parkinson’s disease. The LRRK2 cDNA is ∼7.5 kbp, i.e., too large for AAV and lentivirus vectors. By contrast, the LRRK2 cDNA is readily cloned into a HD CAV-2 or herpes virus vector (Lee et al., 2010). Injection of CAV-LRRK2^G2019S^ into the brain of *M. murinus* induced Parkinson’s disease-like motor symptoms, swelling and loss of neurites, dystrophic neurons, and reduced tyrosine hydroxylase immunoreactivity in the putamen (Mestre-Francés et al., 2018; Lasbleiz et al., 2019).

## Networks, Circuits, Pain, and Behavior

### Networks: Tracing the Relationship Between Input and Output (TRIO)

**FIGURE 3:**
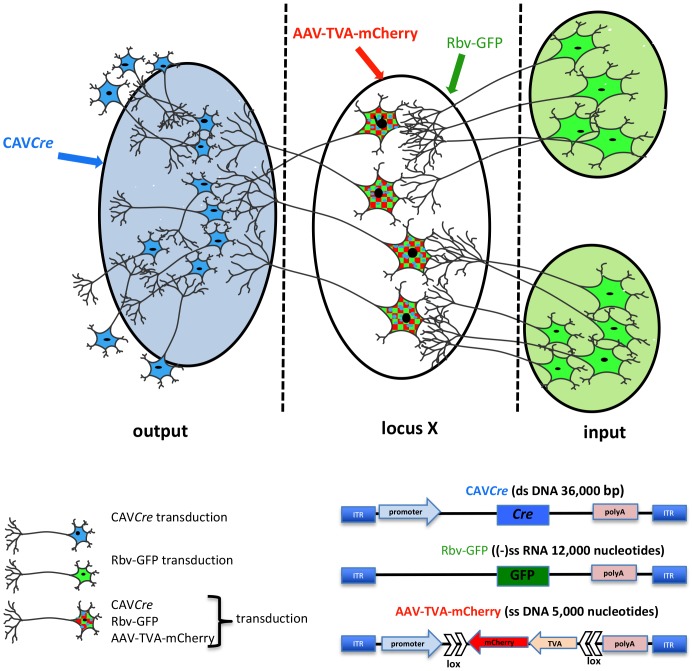
TRIO: tracing the relationship between input and output neural circuits. Three viral vectors are used. CAV-2, a nonenveloped capsid containing an ∼32 kbp double-stranded DNA genome; AAV, a nonenveloped capsid containing a single-stranded DNA genome of ∼5,000 nucleotides: and a rabies virus, an enveloped particle containing negative single-stranded RNA genome. TRIO takes advantage of the retrograde transport capacity of CAV-2 vectors, the local transduction of neurons without significant retrograde transport of the AAV vector, and the transsynaptic transport of rabies vector. In this schema, CAV*Cre* (in blue) and AAV-TVA-mCherry (in red), containing the receptor for the rabies virus (TVA) and mCherry flanked by double inverted lox sequences, are injected in the respective locations (shaded blue for CAV*Cre*, locus X/unshaded oval for the AAV). AAV vectors need ∼2 weeks to generate the second strand of their genome. It is then that Cre expression induces the expression the rabies virus receptor by flipping the expression cassette to “on.” All mCherry^+^ cells have been transduced by CAV*Cre* and AAV-TVA-mCherry, which identifies the output connection of locus X. Rbv-GFP is then injected at the same coordinates of the AAV injections (locus X unshaded oval). Only mCherry positive cells, which also express TVA for the pseudo-typed rabies vector can be transduced, therefore mCherry^+^ and GFP^+^ cells have been transduced by all three vectors. Rbv-GFP then undergoes a round of replication, and new particles are transported into neurons that synapse to the infected cell. GFP^+^/mCherry^−^ cells identify input regions.

Deciphering how neural circuits are anatomically organized with respect to input and output connections is instrumental in understanding how the brain processes information. TRIO is a technique to map input–output connections in a selected region (Beier et al., 2015; Schwarz et al., 2015). To trace neural pathways, TRIO uses the combinatorial power of CAV-2, AAV, and rabies virus vectors (Figure 3). Briefly, an AAV2 vector, which preferentially infects cells at the site of injection, contains genes for an engineered receptor for a pseudo-typed rabies virus and a fluorescent protein (e.g., mCherry). The open reading frames for the receptor and mCherry in the AAV vector are in reverse orientation (3′–5′) and flanked by double-inverted oriented (DIO) lox sequences (i.e., a DIO cassette). Thus, an AAV-infected neuron must contain Cre recombinase for the receptor and mCherry to be expressed. Cre is delivered by CAV*Cre*, which is injected at a site that may contain axonal projection of the neurons infected by the AAV vector. This combination allows one to identify the output of the neurons in the locus/region/structure targeted by the AAV injections, because these cells become mCherry^+^. To identify which cells synapse to the AAV and CAV*Cre* infected neurons (which now express the receptor for the pseudo-typed rabies virus also), one injects the rabies vector that harbors a GFP expression cassette. Here, infection is restricted to the subset of cells expressing its receptor, i.e., only cells infected with both CAV*Cre* and AAV vectors. The rabies vector turns these cells yellow (mCherry + GFP = yellow), and is capable of a single replication cycle, transcytosis, and retrograde transport to synapsing neurons. Cre^+^/mCherry^+^/GFP^+^ cells are infected by the three vectors, while GFP^+^ cells synapse to the neurons at the injection site. TRIO, as well as a more complex version called cTRIO (Beier et al., 2015; Schwarz et al., 2015), allows identification of the relationship between input and output circuits, thereby improving our understanding of normal brain function and potentially diseases like depression, addiction, and schizophrenia.

### Functional Characterization of Circuits

When an anatomical circuit has been identified and/or characterized it can be targeted for functional analyses. Previously, the complexity of neural anatomy, i.e., many thousands of neurons synapsing with thousands of other neurons, made identifying the involvement of a given neuronal pathway in a specific behavior a daunting challenge. Combining optogenetics (e.g., channelrhodopsins, a family of proteins that function as light-gated ion channels) (Adamantidis et al., 2015), DREADDs *(*designer receptor exclusively activated by designer drugs) (Roth, 2016) and retrograde vector transport allows specific targeting and manipulation of neural pathways. In turn, these tools have improved our understanding of how the activity of specific neuronal pathways can act as determinants of behavior (Sara, 2009; Takeuchi et al., 2016). Often, optogenetic and DREADD technologies are used in knock-in mice expressing Cre, which limits their applicability. As suggested by Nair et al. (2013), using these technologies together with Cre-expressing viral vectors, e.g., CAV-2, provides a way to target specific neural pathways with cellular resolution. These options include (1) combining CAV*Cre* and a second viral vector containing a Cre-inducible (DIO) DREADD/channelrhodopsin expression cassette, (2) using CAV*Cre* in knock-in mice with a DIO DREADD/channelrhodopsin expression cassette, or (3) injecting CAVFlexFlp vector harboring a Flippase-inducible Ce expression cassette into a knock-in mouse expressing DIO DREADD/channelrhodopsin in a subset of neurons (e.g., under transcriptional control) (Beier et al., 2015; Schwarz et al., 2015). Any of these approaches would allow the activation of a group of neurons in one area of the nervous system that innervate a distal area.

## Could Cav-2 Vector Infection Perturb Neuron Function?

One way to measure the influence of viral vectors on tissue homeostasis is to characterize the changes in the transcriptional signature. This approach helps dissect the interplay between vectors and host-specific cells, predict vector impact, and identify vector-specific responses (Piersanti et al., 2013, 2015). Global transcriptional analyses were used to analyze the impact of E1/E3-deleted and HD vectors on human cells (Simão et al., 2016). DNA chips were used to assess the transcriptional changes in lung fibroblasts induced by HAd5 compared to AAV5 particles (Stilwell et al., 2003; Stilwell and Samulski, 2004). Furthermore, transcriptome analyses corroborate *in vivo* data indicating that AAVs have a quantifiably different effect on host cells compared HAds. Such analyses also showed that the activation of the innate response by group C HAdVs is affected by HAd-factor X (FX) complexes (Doronin et al., 2012).

**BOX 2 |** Take home messages.

• CAV-2 vectors preferentially transduce neurons in the rodent, canine, and primate brain due to the neuronal expression of CAR in the brain parenchyma.
• Uptake at axons and delivery to the somata (retrograde transport) occurs via pH neutral multitasking endosomal vesicles.
• Previously cumbersome, CAV-2 vector cloning, generation and production are now straightforward due to SLiCE and I-Sce1-ER expression in transcomplementing cells.
• The payload/cloning capacity of E1/E3-deleted CAV-2 vectors is >7 kbp.
• Transgene expression from E1/E3-deleted CAV-2 vectors is stable for at least 6 months in rats.
• The payload/cloning capacity of HD CAV-2 vectors is > 30 kbp.
• Transgene expression from HD CAV-2 vectors is stable for at least 12 months in rodents and in primates.
• When E1/E3-deleted CAV-2 vectors transduce neural precursor or neural stem cells, the cells still efficiently integrate into the existing circuits.
• HD CAV-2 vectors do not significantly perturb the transcriptome of human NPCs or neurons in the NHP brain.
• CAV-2 vectors have been used to: treat mucopolysaccharidoses in the mouse and dog, model Parkinson’s disease in NHPs by expressing a hyperactive kinase form of LRRK2, and used by numerous groups to understand and explore neuronal networks.

**FIGURE 4:**
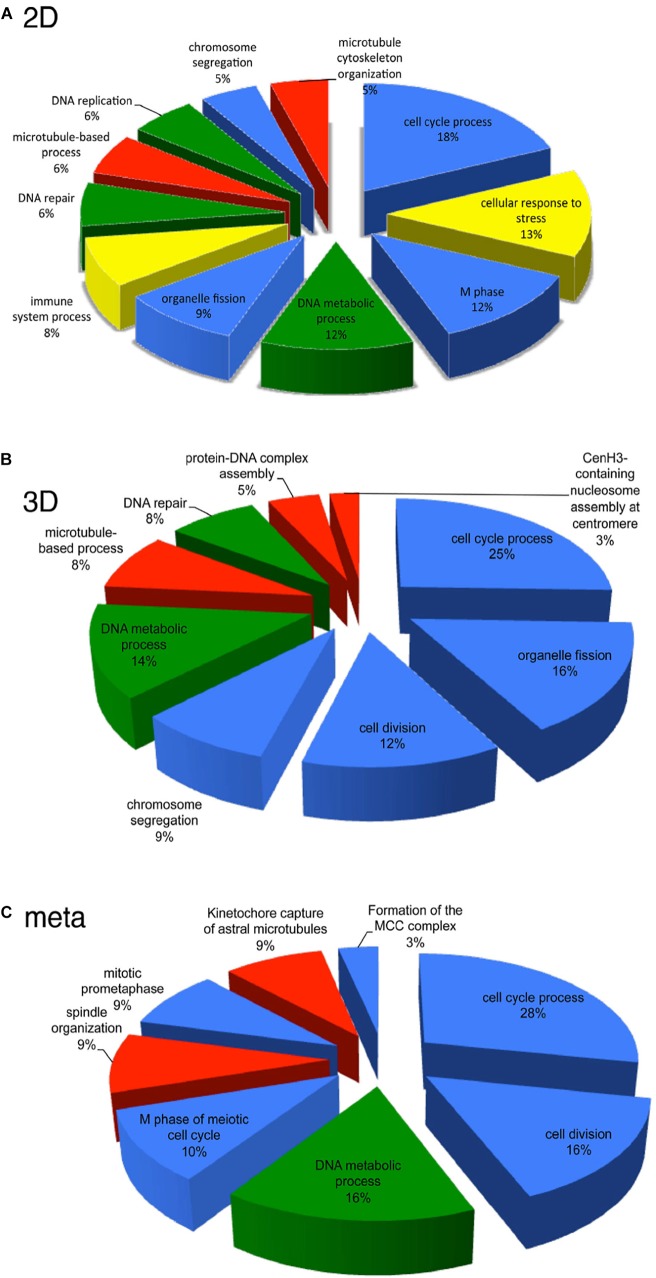
Transcriptional pathways affected by HD-CAV-2 in human neuron *in vitro* and *M. murinus* neurons *in vivo.* **(A,B)** Functional transcriptional signature of HD-CAV-2 in classic 2D cultures **(A)** or 3D bioreactor cultures **(B)** from differentiated human midbrain progenitor cells obtained by *in silico* G-profiler analysis of microarray data on RNA extracted at 5 days post-vector addition. **(C)** Meta-analysis of functional signature of HD-CAV-2 in 2D or 3D cultures on RNA extracted at 5 days post-vector addition and of RNA extracted from *M. murinus* striatum at 24 h and 28 days after HD-CAV-2 stereotaxic injection. Green – DNA metabolism related functions; red – microtubule related processes; yellow – immune response related processes; blue – cell division/organization related functions.

Given the potential of HD CAV-2, DNA chips were used to characterize the influence of HD CAV-2, HD HAd5, and lentivirus vectors on transcription in human midbrain neuroprogenitor cells differentiated into dopaminergic neurons and propagated in 2D cultures (Piersanti et al., 2013, 2015; Mestre-Francés et al., 2018). The three viral vectors differentially affected the transcriptome, activating pro-survival genes and slightly altering neuron morphogenesis. While HD CAV-2 did not negatively affect neuronal development, it did induce an innate immune response. HD CAV-2 induced a lower transcriptional response when compared to HD HAd5 and lentivirus vectors. The HD CAV-2 transcriptional signature was further refined using 3D human neurospheres. These neurospheres were generated from midbrain progenitors and display multiple characteristics of human brain (Brito et al., 2012; Gualda et al., 2014; Simão et al., 2015). The effect of HD CAV-2 on the transcriptome in neurospheres was similar to that observed in the 2D culture system, with the notable addition of centromeric- and microtubule-related genes in 3D cultures. Finally, DNA chips were used to analyze transcriptional alterations in brain tissue of *M. murinus* following stereotaxic injection of the HD-CAV-2 into the caudate nucleus. The vector induced a modest modulation of genes involved in the immune response, intracellular trafficking and transcriptional regulation. Comparing the data sets from the three model systems (2D and 3D midbrain cultures and *M. murinus* brains) reveals that HD CAV-2 specifically modulates genes related to the cell cycle, microtubule organization and DNA metabolism (Figure 4). We hypothesize that the effects on microtubule-related genes is due to the interaction of the CAV-2 fiber with CAR, while the DNA damage response reflects activation of host DNA-damage repair systems by free viral DNA ends in the transduced cells. The modest effect on genes involved in the immune response could also be related to the immunogenicity of GFP.

## Conclusion

Viral vectors will continue to help advance the characterization of brain function, circuitry and neural plasticity. Our understanding of the capabilities of CAV-2 vectors, their titers, and cloning capacity are assets that will allow users to create multiple and/or complex cassettes under transcriptional or post-translational control to explore the healthy and diseased brain (Box 2).

Nevertheless, there are caveats. As mentioned above, we do not know the transduction efficiency of all neuronal types. Because CAV-2 relies on CAR for uptake and transport, CAR-negative neurons will be poorly transduced. In another report in this Research Topic series, Iria Gonzales Dopeso-Reyes will show that the CAR expression pattern in mice, rats, microcebes and macaques varies considerably. One way to circumvent the lack of CAR expression by some neurons is the approach developed by Li et al. (2018). They used a receptor complementation strategy via an AAV vector to express CAR in candidate projection neurons. Exogenous CAR expression mediated by the AAV vector increased CAV-2 retrograde labeling efficacy. However, a confounding issue is our rudimentary understanding of the neuronal functions of CAR. Exogenous expression of CAR, which can be recruited to activate synapses (Zussy et al., 2016), might affect neuronal function and downstream cognitive assays.

Another approach to increase CAR-tropic virus infection is the use of nanovesicles covered with CAR (marketed by Takara^TM^). The CAR-coated nanovesicles fuse with the plasma membrane of cells to transiently allow CAV-2 attachment and internalization. Whether the CAR-covered nanovesicles can be used in the mammalian brain has not been reported. *In vivo* use of nanovesicles could create complications because all cells would likely take up the microvesicles, thereby obviating the preferential transduction of neurons by CAV-2 vectors. Yet, neuron-specific transgene expression could be controlled by using appropriate promoters driving the expression cassette. If nanovesicles were used to characterize neuronal circuits, the exogenous CAR would need to be trafficked to distal regions of the axons. Finally, exogenous CAR could affect neuronal function and downstream cognitive assays. We found that, after CAR depletion in the striatum, its replacement takes approximately 2 weeks (Zussy et al., 2016). This suggests, but does not directly demonstrate, that CAR is relatively stable and its aberrant expression could have long-term effects on neuron homeostasis.

A third approach is modifying the CAV-2 capsid to preferentially target neuronal subtypes. Numerous methods have been developed to modify the tropism of adenoviruses. These include swapping fiber knobs from other types and adding ligands into the fiber, penton base, hexon, or protein IX by inserting a sequence into their open reading frame. Finally, one could create a “bi-polar” recombinant protein with one end that binds the fiber knob and the other end that targets a cell surface moiety. Several reviews describe these and other approaches (Arnberg, 2012; Yoon et al., 2016; Yamamoto et al., 2017; Zhang and Ehrhardt, 2017).

## Author Contributions

All authors contributed to writing and figure design.

## Conflict of Interest Statement

The authors declare that the research was conducted in the absence of any commercial or financial relationships that could be construed as a potential conflict of interest.
